# The Traditional Uses, Phytochemistry, Pharmacology, Toxicology, and Clinical Uses of *Metagentiana Rhodantha* (Franch.) T.N.Ho and S.W.Liu, an Ethnomedicine in Southwest China

**DOI:** 10.3389/fphar.2021.658628

**Published:** 2021-04-26

**Authors:** Botao Chang, Songjiang Tang, Rong Chen, Nan Xiao, Jingsong Zhu, Mengxian Tian, Huizhong Jiang, Xi Li, Zhonglu Jian, Xu Han, Ying Gao, Qi Yao

**Affiliations:** ^1^ The First Affiliated Hospital of Guizhou University of Traditional Chinese Medicine, Guiyang, China; ^2^ Graduate College, Guizhou University of Traditional Chinese Medicine, Guiyang, China

**Keywords:** Metagentiana rhodantha (Franch.), phytochemistry, pharmacology, toxicity, traditional uses

## Abstract

**Background:** *Metagentiana rhodantha* (Franch.) T.N.Ho and S.W.Liu (MR) belongs to Gentianales*,* and it is often called Hong-hua-long-dan in Chinese. Traditionally, it has been used to cure acute icteric hepatitis, sore throat, dysentery, acute gastritis, carbuncle, and furuncle based on traditional Chinese medicine (TCM) concepts.

**Aim of Study:** This review manages to provide a critical and comprehensive analysis on the traditional uses, phytochemistry, pharmacology, toxicology, and clinical uses of MR and to evaluate the therapeutic potential of this plant.

**Methods:** Relevant data mainly literatures on MR were selected from available database. All the papers reviewed provided evidence that the source herbs were reliably identified.

**Results:** The heat-clearing and removing the phlegm, and purging fire and removing toxicity of MR contribute to its dispelling jaundice, and clearing lung heat and cough. The compounds isolated from this plant include iridoids and secoiridoids, phenolic acids, ketones, triterpenoids, flavonoids, benzophenone glycosides, and others. Mangiferin (MAF) is a characteristic substance from this plant. The pharmacological studies show that some extracts and compounds from MR exhibit anti-inflammatory, antinociceptive, antibacterial, hepatoprotective, cardioprotective, and other effects which are associated with the traditional uses of this plant. The toxicological studies suggest that MAF is less toxic in mice and dogs. Nowadays, Chinese patent drugs such as Feilike Jiaonang and Kangfuling Jiaonang containing MR have been used to cure cough, asthma, chronic bronchitis, dysmenorrhea, and appendagitis.

**Conclusion:** Although the current studies provide related research information of MR, it is still necessary to systemically evaluate the chemistry, pharmacology, toxicity, and safety of the extracts or compounds from this plant before clinical trials in the future. In addition, except for lung infection-related diseases, analgesia, anti-tumor, and hypertriglycemia may be new and prior therapeutic scopes of this ethnomedicine in the future.

## Introduction

There are about 20 genera and 419 species of Gentianaceae in China, of which 2 genera and 251 species are endemic. The vast majority of genera and species are distributed in mountainous areas of southwest China, among which Gentiana, Swertia and Lomatogonium are the most common.

MR is a perennial herb and its common names include long-dan-cao, xiao-qing-yu-dan, xing-xiu-hua, jiu-ri-hua, and jia-jia-shan (The Plant List, 2012). Its basic origin contains MR and *Gentianopsis paludosa* (Hook.f.) Ma. Among the Gentiana species, roots and rhizomes of *G. scabra* Bunge and *G. rigescens* Franch. ex Hemsl., original materials of “Long-dan” are used as hepatoprotective agents in southwest and northeast China.

Conventionally, its whole plant or root is used. MR is summarized as bitter in flavor and cold in nature according to its TCM characteristics. Also, it is attributed to lung and liver meridians (Editorial board of Chinese materia medica and State administration of traditional Chinese medicine, 2005). Its functions include clearing heat and removing dampness, and purging fire and removing toxic substance, which facilitates treating damp-heat jaundice, lung-heat cough, and dysuria.

Nowadays, it has revealed that the compounds or extracts from MR exhibit anti-inflammatory (Somani et al., 2016; Qu et al., 2017), antioxidant (Saha et al., 2016; Mahalanobish et al., 2019), analgesic (Dar et al., 2005; Garrido-Suárez et al., 2014), antitumor (Takeda et al., 2016; Deng et al., 2018; Tang et al., 2019; Xiao et al., 2020), hepatoprotective (Das et al., 2012; Jain et al., 2013), and other effects. However, the pharmacological research mainly focuses on the characteristic compounds especially MAF but not the extracts. Furthermore, the active components in the extracts of MR have not been clearly clarified yet as well as the mechanisms of action. In addition, an appropriate effective dose, the frequency of dosing, or the duration of treatment should be taken into consideration in biological evaluations (Beharry and Heinrich, 2018).

The present review manages to provide a critical and comprehensive analysis on the traditional uses, phytochemistry, pharmacology, toxicology, and clinical uses of MR. Furthermore, the relationship between the traditional uses and the clinical uses of MR is also focused. Based on this, we expect to provide some evidences for the therapeutic value of this plant in clinic in the future.

## Botanical Description

In China, MR is mainly distributed in Yunnan, Sichuan, Guizhou, Gansu, Shaanxi, Henan, Hubei, and Guangxi provinces. It often grows in areas between 570~1750 m above the sea level, mostly on alpine shrubs, grasslands and forests (Flora of China Editorial Committee, The Chinese Academy of Sciences, 1997). MR is a typical alpine plant which likes cold climates and has a strong cold tolerance; Its temperature requirement is not strict. However, it needs suitable temperature and certain light when germination occurs. Meanwhile, hot and humid weather is avoided during seedling.

MR was first included in Chinese Pharmacopoeia as the name of “Hong-hua-long-dan” in 1977. In the 2020 edition, it is included as the Latin name of *Gentiana rhodantha* Franch (Chinese Pharmacopoeia Commission, 2020).

**FIGURE 1:**
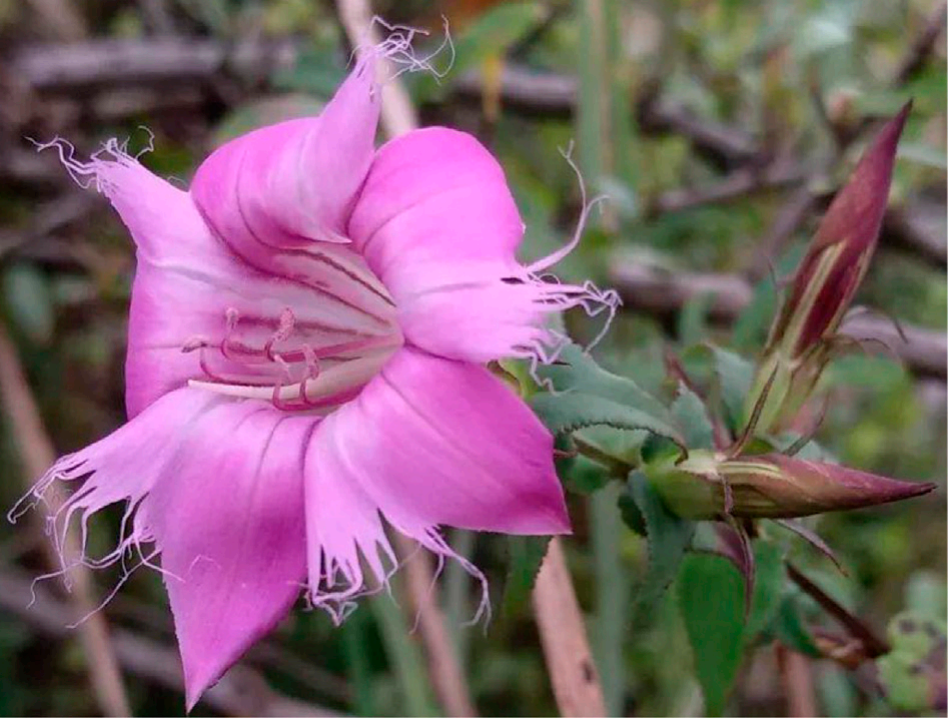
Image of *Metagentiana rhodantha* (Franch.) T.N.Ho and S.W.Liu.

MR is a 20~50 cm tall perennial herb with a short rhizome. Stems are erect; branches are spread and glabrous; petioles are 5~10 mm in length; leaf blades (2~4 × 0.7~2 cm) are elliptic, ovate, or obovate, and they have narrowed base, serrulate margin, distinct midvein, and acute apex. Stem leaves are spaced widely; leaf blades (1~3 × 0.5~2 cm) are cordate, ovate-triangular, or broadly ovate, and shorter than internodes; Flowers are solitary, sessile, and terminal (Figure 1) (Flora of China, http://www.efloras.org/florataxon.aspx?flora_id=2&taxon_id = 200018063).

Purple calyx tube (0.7~1.3 cm) is membranous, narrowly winged, narrowly obconic; lobes are linear-lanceolate and 5~10 mm, and they have outside prominent midvein, ciliolate margin, and acuminate apex. Pale purple corolla has blackish streaks that are tubular to funnelform and 2.5~4.5 cm in length. Lobes (5~9 mm) are ovate to ovate-triangular. triangular plicae (4~5 mm) are apex fringed. Unequal stamens grow at basal part of corolla tube; filaments are 5~12 mm anthers (2.5~3 mm) are narrowly ellipsoid. Style is 6~8 mm; stigma lobes are linear. Capsules (2~2.5 cm) are ellipsoid; Light brown seeds are subglobose, winged, and approximately 1 mm in diameter. Flower and fruit periods are from October to February of the following year (Flora of China, http://www. efloras. org/florataxon. aspx?flora_id = 2 and taxon_id = 200018063). Until now, no report has been reported on chemical components from fruits of MR.

Currently, MR is cultivated sporadically by the residents in southwest China. MAF is a characteristic substance of MR (Luo et al., 2014; Kang et al., 2016; Liu et al., 2020). However, the contents of MAF in MR from different production area or different parts of this plant are varied (Luo et al., 2014). Thus, it is necessary to establish a quality standard for this medicinal component for the clinical use of this plant.

## Traditional Uses

MR is respectively called “Qing-ye-dan” (Bai nation), “Bo-long” (Dong nation), “Wo-du-zi-lan-mo” (Lisu nation), and “Ming-bu-ju-jiao” (Miao nation) (Qiu and Du, 2005).

Traditionally, MR is widely used by the minor nations including Bai, Dong, Lisu, and Miao nations in southwest China. In concepts of these minor nations, the whole plant or root is used to treat acute icteric hepatitis, sore throat, dysentery (whole plant in decoction in Yunnan) (Bai nation) (whole plant in decoction in Guangxi) furuncle (Dong nation) (whole plant in decoction in Guizhou) lung heat cough, jaundice, stomachache, hematochezia, infantile convulsion, scald (Lisu nation) (whole plant in decoction in Guizhou) carbuncle and acute gastritis (Miao nation) (Table 1).

**TABLE 1:** Traditional uses of MR in China.

No	Component(s)	Traditional uses	Usage(s)	Reference(s)
1	Certain amount of MR	Curing swelling and pain of eye, jaundice, and furuncle	Decoction and take orally (2.5–15 g a day)	Guangxi health administration (1959)
2	Certain amount of MR	Curing acute and chronic hepatitis, cholecystitis, conjunctivitis, sore throat, and hypertension	Decoction and take orally (15–25 g a day)	The minister of health of logistics department of Kunming military command (1970)
3	Fresh MR 3060 g and an amount of pork	Curing wind-heat cough	Stewing and take soup and meat orally	Qiu and Du (2005)
4	MR root 150 g	Curing rheumatism	Immerged in alcohol and take orally	Qiu and Du (2005)
5	MR 15 g, *Polygonatum odoratum* (mill.) druce 9 g	Curing ascarid	Stewing with rice and take orally for 2 times	Qiu and Du (2005)
6	MR 10 g, *Chrysanthemum × morifolium* (ramat.) hemsl. 5 g, *Lonicera japonica* thunb. 5 g	Curing acute icteric hepatitis, carbuncle and furuncle	Decoction and take orally	Qiu and Du (2005)
7	MR 16 g, *Viscum diospyrosicola* hayata 16 g	Curing whooping cough	Decoction and take orally for 3 days	Qiu and Du (2005)
8	MR 10 g, *Selaginella moellendorffii* hieron. 10 g, sweet ferment rice 31 g	Curing cough with bloody sputum	Steaming and take orally	Qiu and Du (2005)
9	MR 31 g	Curing hemorrhagic gonorrhea	Decoction and take orally for three times	Qiu and Du (2005)
10	MR 16 g	Curing throat sore	Decoction and take orally slowly for several times a day	Qiu and Du (2005)

The full taxonomic names of the species have been validated using https://mpns.science.kew.org/mpns-portal/.

MR has been used to treat acute and chronic hepatitis and cholecystitis, acute conjunctivitis, sore throat, hypertension, poor appetite (whole plant in decoction in Yunnan) (The minister of health of logistics department of Kunming military command, 1970), *Streptococcus pneumoniae*-induced pneumonia (aqueous extract from whole plant) (Wang et al., 2018), pain (MAF) (Dar et al., 2005; Garrido-Suárez et al., 2014), tumors (MAF) (Takeda et al., 2016; Deng et al., 2018; Mu et al., 2018), liver injury (MAF) (Das et al., 2012; Jain et al., 2013).

## Phytochemistry

**FIGURE 2:**
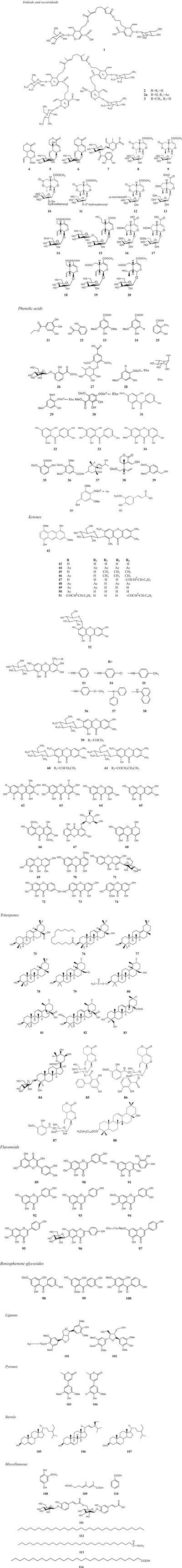
Isolated and synthesized compounds from MR.

Currently, phytochemistry studies on MR have isolated and synthesized 114 compounds including 20 iridoids and secoiridoids (**1**~**20**), 21 phenolic acids (**21**~**41**), 33 ketones (**42**~**74**), 14 triterpenes (**75**~**88**), 9 flavonoids (**89**~**97**), 3 benzophenone glycosides (**98**~**100**), 2 lignans (**101, 102**), 2 pyrones (**103, 104**), 3 sterols (**105**~**107**), 7 miscellaneous compounds (**108**~**114**). These compounds were mainly isolated from the whole plant or the root of this plant (Table 2; Figure 2).

**TABLE 2:** The isolated and synthesized compounds from MR.

No	Name	Parts of plant	Source	Reference(s)
—	*Iridoids and secoiridoids*			
1	Rhodenthoside A	Whole plant	Methanol extract	Ma et al. (1996)
2	Rhodenthoside B	Whole plant	Methanol extract	Ma et al. (1996)
3	Rhodenthoside C	Whole plant	Methanol extract	Ma et al. (1996)
4	Sweroside	Whole plant	Methanol extract	Ma et al. (1996); Pan et al. (2015)
5	Swertiamarin	Whole plant	Methanol extract	Ma et al. (1996); Pan et al. (2015)
6	Gentiopicroside	Whole plant	Methanol extract	Pan et al. (2015)
7	2’-(2,3-dihydroxybenzoyl)-sweroside	Whole plant	Methanol extract	Yao et al. (2013)
8	Kingiside	Whole plant	Methanol extract	Xu et al. (2008)
9	8-Epikingiside	Whole plant	Methanol extract	Xu et al. (2008)
10	2′-*O*-(3″-hydroxybenzoyl)-8-epikingiside	Whole plant	Methanol extract	Xu et al. (2008)
11	2′-*O*-(3″-hydroxybenzoyl)-kingiside	Whole plant	Methanol extract	Xu et al. (2008)
12	6′-*O*-*p*-coumaroyl-8-epikingiside	Whole plant	Methanol extract	Xu et al. (2008)
13	Loganic acid 11-*O*-*β*-glucopyranosyl ester	Whole plant	Methanol extract	Xu et al. (2008)
14	6′-*O*-*β*-glucopyranosyl secologanoside	Whole plant	Methanol extract	Xu et al. (2008); Luo et al. (2018)
15	6′-*O*-*β*-glucopyranosyl secologanol	Whole plant	Methanol extract	Xu et al. (2008)
16	Loganic acid	Whole plant	Methanol extract	Xu et al. (2008)
17	6′-*O*-*β*-d-glucopyranosyl loganic acid	Whole plant	Methanol extract	Xu et al. (2008)
18	Secologanoside	Whole plant	Methanol extract	Xu et al. (2008)
19	Secoxyloganin	Whole plant	Methanol extract	Xu et al. (2008); Luo et al., 2018
20	Alpigenoside	Whole plant	Methanol extract	Xu et al. (2008)
—	*Phenolic acids*			
21	Gallic acid ethyl ester	Whole plant	Ethanol extract	Chen et al. (2013)
22	Salicylic acid	Whole plant	Ethanol extract	Chen et al. (2013)
23	Syringic acid	Whole plant	Ethanol extract	Chen et al. (2013)
24	Vanillic acid	Whole plant	Ethanol extract	Chen et al. (2013)
25	3-Hydroxy-2-methoxy benzoic acid	Whole plant	Methanol extract	Xu et al. (2011)
26	2-Hydroxy-3-*O*-*β*-D-glucosyloxy benzoic acid methyl ester	Whole plant	Methanol extract	Xu et al. (2011)
27	Syringic acid-4-*O*-*α*-l-rhamnoside	Whole plant	Methanol extract	Xu et al. (2011)
28	1,2-Dihydroxy-4-methoxybenzene 1-*O*-*α*-*L*-Rhamnopyranosyl-(1→6)-*β*-d-glucopyranoside	Whole plant	Methanol extract	Xu et al. (2011)
29	1,2-Dihydroxy-4,6-dimethoxybenzene 1-*O*-*α*-*L*-Rhamnopyranosyl-(1→6)-*β*-d-glucopyranoside	Whole plant	Methanol extract	Xu et al. (2011)
30	Methyl 2-*O*-*β*-d-glucopyranosyl-2,4,6-trihydroxybenzoate	Whole plant	Methanol extract	Xu et al. (2011)
31	Rhodanthenone A	Whole plant	Methanol extract	Xu et al. (2011)
32	Rhodanthenone B	Whole plant	Methanol extract	Xu et al. (2011)
33	Rhodanthenone C	Whole plant	Methanol extract	Xu et al. (2011)
34	Rhodanthenone D	Whole plant	Methanol extract	Xu et al. (2011)
35	2-(*β*-D-glucopyranosyloxy)-3-hydroxybenzoic acid	Whole plant	Methanol extract	Xu et al. (2011)
36	Glucosyringic acid	Whole plant	Methanol extract	Xu et al. (2011)
37	Alangionoside O	Whole plant	Methanol extract	Xu et al. (2011)
38	1-*O*-*β*-d-glucopyranosyl-4-epiamplexine	Whole plant	Methanol extract	Xu et al. (2011)
39	Vanilloloside	Whole plant	Methanol extract	Xu et al. (2011)
40	Rhyncoside D	Whole plant	Methanol extract	Xu et al. (2011)
41	Ferulic acid	Whole plant	Methanol extract	Pan et al. (2016)
—	*Ketones*			
42	Bellidifolin	Whole plant	Methanol extract	Pan et al. (2016)
43	Mangiferin (MAF)	Whole plant	Methanol extract	Xu et al. (2011); Yao et al. (2015)
44	2-*β*-d-tetraacetoxyglucopyranosyl-1.3,6,7-tetraacetoxy-9*H*-Xanthen-9-one	—	Based on MAF	Dar et al. (2005); Faizi et al. (2006)
45	2-*β*-d-glucopyranosyl-1-hydroxy-3,6,7-trimethoxy-9*H*-Xanthen-9-one	—	Based on MAF	Dar et al. (2005); Faizi et al. (2006)
46	2-*β*-d-tetraacetoxyglucopyranosyl-1-hydroxy-3,6,7-Trimethoxy-9*H*-xanthen-9-one	—	Based on MAF	Dar et al. (2005); Faizi et al. (2006)
47	2-*β*-d-glucopyranosyl-7-cinnamoyloxy-1,3,6-trihydroxy-9*H*-xanthen-9-one	—	Based on MAF	Dar et al. (2005); Faizi et al. (2006)
48	2-*β*-d-tetraacetoxyglucopyranosyl-1,6,7-tetraacetoxy-3-Hydroxy-9*H*-xanthen-9-one	—	Based on MAF	Dar et al. (2005); Faizi et al. (2006)
49	2-*β*-d-tetraacetoxyglucopyranosyl-1-acetoxy-3,6,7-Trihydroxy-9*H*-xanthen-9-one	—	Based on MAF	Dar et al. (2005); Faizi et al. (2006)
50	2-*β*-d-tetraacetoxyglucopyranosyl-1.3,6,7-tetraacetoxy-9*H*-Xanthen-9-one	—	Based on MAF	Dar et al. (2005); Faizi et al. (2006)
51	2-*β*-d-tetracinnamoyloxyglucopyranosyl-7-cinnamoyloxy-1,3,6-trihydroxy-9*H*-xanthen-9-one	—	Based on MAF	Dar et al. (2005); Faizi et al. (2006)
52	4-*β*-d-glucopyranosyl-1.3,6,7-tetrahydroxy-9*H*-xanthen-9-one	—	Based on MAF	Dar et al. (2005); Faizi et al. (2006)
53	5-(N-phenylamino methyleno) mangiferin	—	Based on MAF	Kant et al. (2011)
54	5-(N-*p*-chlorophenylamino methyleno) mangiferin	—	Based on MAF	Kant et al. (2011)
55	5-(N-4-methyl phenylamino methyleno) mangiferin	—	Based on MAF	Kant et al. (2011)
56	5-(N-*p*-methoxy phenylamino methyleno) mangiferin	—	Based on MAF	Kant et al. (2011)
57	5-(N-N-diphenylamino methyleno) mangiferin	—	Based on MAF	Kant et al. (2011)
58	5-(N-α-napthylamino methyleno) mangiferin	—	Based on MAF	Kant et al. (2011)
59	7.2′,3′,4′,6′-penta-acetyl-mangiferin	—	Based on MAF	Li et al. (2013)
60	3,6,7,2′,3′,4′,6′-hepta-propionyl-mangiferin	—	Based on MAF	Li et al. (2013)
61	3,6,7,2′,3′,4′-hexa-butyryl-mangiferin	—	Based on MAF	Li et al. (2013)
62	Lancerin	Whole plant	Methanol extract	Xu et al. (2011)
63	Neomangiferin	Whole plant	Methanol extract	Xu et al. (2011)
64	1.3,7,8-Tetrahydroxylxanthone	Whole plant	Ethanol extract	Chen et al. (2013)
65	1.3,6,7-Tetrahydroxylxanthone	Whole plant	Ethanol extract	Chen et al. (2013)
66	1,3,7-Trihydroxy-4,8-dimethoxyxanthone	Whole plant	Ethanol extract	Chen et al. (2013)
67	Norswertinaolin	Whole plant	Ethanol extract	Chen et al. (2013)
68	1.3,5,8-Tetrahydroxylxanthone	Whole plant	Methanol extract	Yao et al. (2013); Yao et al. (2015)
69	1.2,6,8-Tetrahydroxylxanthone	Whole plant	Methanol extract	Yao et al. (2013)
70	1,3,8-Trihydroxyxanthone-5-*O*-*β*-D-glycoside	Whole plant	Methanol extract	Yao et al. (2013); Yao et al. (2015)
71	1,3,6-Trihydroxyxanthone-2-*C*-*β*-D-glycoside	Whole plant	Methanol extract	Yao et al. (2013)
72	1,3,7-Trihydroxyxanthone-2-*C*-*β*-D-glycoside	Whole plant	Methanol extract	Yao et al. (2015)
73	Norswertianin	Whole plant	Methanol extract	Yao et al. (2013); Yao et al. (2015)
74	Triptexanthoside A	Whole plant	Methanol extract	Yao et al. (2015)
—	*Triterpenes*			
75	Oleanolic acid	Whole plant	Methanol extract	Yao et al. (2013)
76	Erythrodiol 3-*O*-palmitate	Whole plant	Ethanol extract	Chen et al. (2013)
77	*α*-amyrin	Whole plant	Ethanol extract	Chen et al. (2013)
78	*β*-amyrin	Whole plant	Methanol extract	Yao et al. (2013)
79	Ursolic aldehyde	Whole plant	Ethanol extract	Chen et al. (2013)
80	Uvaol 3-*O*-acetoxy	Whole plant	Ethanol extract	Chen et al. (2013)
81	Ursolic acid	Whole plant	Ethanol extract	Chen et al. (2013)
82	2*α*-hydroxyursolic acid	Whole plant	Ethanol extract	Chen et al. (2013)
83	Epihedaragenin	Whole plant	Methanol extract	Yao et al. (2013)
84	Gentirigeosides A	Whole plant	Methanol extract	Yao et al. (2013)
85	Anarogentin	Whole plant	Methanol extract	Pan et al. (2016)
86	Macrophylloside B	Whole plant	Methanol extract	Pan et al. (2016)
87	Rindoside	Whole plant	Methanol extract	Pan et al. (2016)
88	Erythrodiol 3-palmitate	Whole plant	Ethanol extract	Chen et al. (2013)
—	*Flavonoids*			
89	Quercetin	Whole plant	Ethanol extract	Chen et al. (2013)
90	Isoorientin	Whole plant	Methanol extract	Yao et al. (2013); Yao et al. (2015)
91	3,5,7,2′,3′,4′-hexahydroxyflavone	Whole plant	Methanol extract	Yao et al. (2013)
92	Luteolin	Whole plant	Methanol extract	Yao et al. (2013); Yao et al. (2015)
93	Naringerin	Whole plant	Methanol extract	Yao et al. (2013); Yao et al. (2015)
94	Lutonarin	Whole plant	Methanol extract	Yao et al. (2013); Yao et al. (2015)
5	Isovitexin	Whole plant	Methanol extract	Yao et al. (2015)
96	Apigenin-6-*C*-*β*-D-glycoside	Whole plant	Methanol extract	Yao et al. (2013)
97	Apigenin-7-*O*-*β*-d-glucopyrosyl-(1–3)-*β*-d-glucopyrosyl-(1–3)-*β*-D-glycoside	Whole plant	Methanol extract	Xu et al. (2011); Yao et al. (2013)
—	*Benzophenone glycosides*			
98	Rodanthenone A	Whole plant	Methanol extract	Xu et al. (2011)
99	Rodanthenone B	Whole plant	Methanol extract	Xu et al. (2011)
100	Rodanthenone C	Whole plant	Methanol extract	Xu et al. (2011)
—	*Lignans*			
101	(-)-Syringaresinol *O*-*β*-d-glucopyranoside	Whole plant	Methanol extract	Xu et al. (2011)
102	Foliachinenoside C	Whole plant	Methanol extract	Xu et al. (2011)
—	*Pyrones*			
103	6-Methyl-4-(4-hydroxy-3,5-dimethoxypheny)-*α*-pyrone	Whole plant	Methanol extract	Yao et al. (2013); Yao et al. (2015)
104	6-Methyl-4-(4-hydroxy-3-dimethoxypheny)-*α*-pyrone	Whole plant	Methanol extract	Yao et al. (2013); Yao et al. (2015)
—	*Sterols*			
105	*β*-sitosterol	Whole plant	Methanol extract	Yao et al. (2013)
106	Stigmasterol	Whole plant	Methanol extract	Yao et al. (2013)
107	*β*-daucosterol	Whole plant	Methanol extract	Yao et al. (2013)
—	*Miscellaneous*			
108	2-Methoxy-1,4-benzenediol	Whole plant	Methanol extract	Yao et al. (2013)
109	2,4-Dimethyl-2,4-diene pentanedioic acid	Whole plant	Methanol extract	Yao et al. (2013)
110	Benzoicacid	Whole plant	Methanol extract	Yao et al. (2013)
111	Scrocaffeside B	Whole plant	Methanol extract	Yao et al. (2013)
112	n-hentriacontane	Whole plant	Ethanol extract	Liu et al. (1985)
113	n-dotriacontanoic acid ethyl ester	Whole plant	Ethanol extract	Liu et al. (1985)
114	n-dotriacontanoic acid	Whole plant	Ethanol extract	Liu et al. (1985)

### Iridoids and Secoiridoids

Iridoids are a class of chemical substances that belong to monoterpenes. Usually, they form iridoid glycosides with glucose in plants. They are roughly classified into iridoid glycosides, 4′-demethyl iridoid glycosides, and secoiridoid glycosides. Secoiridiods are characteristic substances of MR, while their contents are very low. Studies have indicated that secoiridiod glycosides possess wide biological activities such as anti-inflammatory and immunoregulatory (Saravanan et al., 2014a; Saravanan et al., 2014b; Wang et al., 2019a), analgesic (Liu et al., 2016; Garrido-Suárez et al., 2020), anti-tumor (Tang et al., 2019; Xiao et al., 2020) hepatoprotective (Shao et al., 2017; Wu et al., 2017), hypoglycemic (Vaidya et al., 2013a; Dhanavathy, 2015; Vaidya et al., 2012; Vaidya et al., 2009), neuroprotective (Wang et al., 2019b; Wu et al., 2019) effects.

Currently, about 20 iridoids secoiridoids **(1**~**20)** have been isolated from MR (Ma et al., 1996; Xu et al., 2008; Yao, 2013; Pan et al., 2015). Among them, six new iridoidal glucosides **(10**~**15)** were isolated from this plant for the first time (Xu et al., 2008). Their structures were elucidated by spectroscopic analysis in combination with chemical methods. Their chemical structures and names are displayed in Figure 2 and Table 2.

### Phenolic Acids

Phenolic acids are a class of organic acids including *p*-hydroxybenzoic acid, dihydroxybenzoic acid and trihydroxybenzoic acid. It has been reported that phenolic acids have anti-inflammatory, antioxidant (Wang et al., 2020), anti-tumor (Assumpção et al., 2020), anti-bacterial (deOliveira et al., 2021), hepatoprotective (Hassan and Tariq, 2020), cardiovascular protective (Shi et al., 2019) properties. 21 phenolic acids (**21**~**41**) have been separated from the whole plant of MR and their structures are validated by using detailed spectroscopic analyses and chemical methods (Xu et al., 2011; Chen et al., 2013). Their chemical names and structures are listed in Table 2 and Figure 2.

### Ketones

Ketones are compounds in which carbonyl groups are connected to two hydrocarbon groups. According to difference in the hydrocarbon group, ketones are classified into aliphatic ketones, alicyclic ketones, aromatic ketones, saturated ketones, and unsaturated ketones. Until now, 33 ketones (**42**~**74**) have been isolated or synthesized from the whole plant of MR or based on MAF (Xu et al., 2011; Chen et al., 2013) (Table 2; Figure 2).

MAF **(43)** is a characteristic ketone substance isolated from MR (Wu et al., 2010; Luo et al., 2014; Kang et al., 2016; Liu et al., 2020). Luo et al. (2014) established HPLC fingerprint chromatograms of MR by detecting various batches of MR samples in Guizhou. It revealed 40 common peaks with similarities of over 0.97 in the fingerprint chromatograms among 12 batches of the MR samples by selecting MAF as a reference substance.

Also, it has identified 17 common peaks with a similarity of 0.589~0.993 among 11 batches of MR samples by selecting MAF as a reference substance (Liu et al., 2020). Further, the MAF contents were determined in 12 batches of MR samples from different production areas including Yunnan, Guizhou, and Sichuan by using TLC in combination with HPLC. The result showed that the contents of MAF in the samples were from 0.7 to 4.4% (average 2.8%) (Wu et al., 2010). Further, UV-Vis and UPLC fingerprint combined with multivariate analysis were used to study variations in chemical compounds from roots, steams, leaves, and flowers of MR (Shen et al., 2016). The UV-Vis spectra of the roots, stems, leaves and flowers showed certain fingerprint characteristics; The similarity analysis of UPLC fingerprint showed that the chemical components in the leaves were similar to those in the flowers as well as the similarity between the roots and the stems. The similarity coefficients of UPLC fingerprints for the roots from different parts varies widely. Contents of loganic acid and MAF were relative higher in the leaves and flowers [(1.46 ± 0.42) and (51.59 ± 15.45) mg/g], and sweroside content was higher in the roots [(4.41 ± 3.24) mg/g]. Generally, the chemical components were higher in the leaves. Further, loganic acid, MAF, and sweroside contributed significantly to the differences in various parts of this plant. Cluster analysis demonstrated that the chemical compositions in MR from different production areas showed some certain geographic characteristics. In summary, these studies above provided stable and efficient methods to evaluate the quality of MR more systemically.

In addition, 18 MAF derivatives **(44**~**61)** including acyl, methyl, esterified compounds were synthesized and their chemical structures were identified by MS, (1)H, (13)C NMR, 2D NMR and other chemical analytic methods (Dar et al., 2005; Faizi et al., 2006; Kant et al., 2011; Li et al., 2013).

### Triterpenoids

Triterpenoids are substances composing several isoprene groups connecting end to end after removing hydroxyl groups. Most of them are terpenoids possessing 30 carbon atoms, and a small number of them contain 27 carbon atoms. Also, triterpenoids are characteristic substances of MR, and their contents in this plant are few as well as secoiridoids. It has been documented that terpenoids have anti-inflammatory (Sun et al., 2018), antioxidant (Qiu et al., 2016), anti-tumor (Ren and Kingdom, 2019), and anti-bacterial (Yu et al., 2019) effects.

Fourteen triterpenes (**75**~**88**) have been separated from the whole plant of MR and then elucidated by detailed spectroscopic analysis combined with chemical methods (Chen et al., 2013; Yao, 2013). Their chemical structures and names are shown in Figure 2 and Table 2.

### Flavonoids

Originally, flavonoids are a class of compounds derived from 2-phenylchromone. Now, they are thought to be a series of compounds formed by connecting two benzene rings through three carbon atoms, that is, the general term of a class of compounds with C6-C3-C6 structures. Flavonoids are characteristic substances of MR. Studies have suggested that flavonoids have anti-inflammatory (Zaragozá et al., 2020), analgesic (Tian et al., 2019), anti-tumor (Stefanowicz-Hajduk et al., 2020), anti-myocardial ischemic (Zhang et al., 2017), anti-atherosclerotic (Kim and shim, 2019; Wei et al., 2020), and hypoglycemic (Luo et al., 2019) properties.

Currently, 9 flavonoids (**89**~**97**) (Xu et al., 2011; Chen et al., 2013; Yao, 2013; Yao et al., 2015) have been isolated from the whole plant of MR. Besides quercetin **(89)**, isoorientin **(90)** and other flavonoids and flavonoid glycosides were separated from this plant (Chen et al., 2013). The chemical structures were elucidated by detailed spectroscopic analysis in combination with chemical methods (Chen et al., 2013; Yao et al., 2015). Their chemical structures and names are shown in Figure 2 and Table 2.

### Benzophenone Glycosides

Three benzophenone glycosides (**98**~**100**) including two new rare a-pyrone (-2H-pyran-2-one) derivatives, rhodanthpyrones A and B **(98, 99)** were isolated from the whole plants of MR and then their structures were elucidated by using IR, HR-ESI-MS, and 1D- and 2D-NMR analyses (Yao et al., 2015) (Figure 2; Table 2).

### Miscellaneous

Two lignans (**101, 102**) (Xu et al., 2011), 2 pyrone (**103, 104**) (Yao et al., 2015), 3 sterols (**105**~**107**) (Yao, 2013), and 7 miscellaneous compounds (**108**~**114**) (Yao, 2013) were isolated from the whole plant of MR and their chemical structures and names are displayed in Table 2 and Figure 2.

## Pharmacological Effects

Pharmacological effects of the compounds and extracts from MR have been investigated. The pharmacological effects are displayed in Table 3.

**TABLE 3:** Pharmacological effects of extracts and compounds from MR.

Effects	Active components/compounds	Source	Reference(s)
Anti-inflammatory effects	MAF	—	Qu et al. (2017); Somani et al. (2016); Wang et al. (2017a); Mendiola et al. (2020)
	SWM	—	Saravanan et al. (2014a); Saravanan et al. (2014b); Wang et al. (2019a)
	Gent	—	—
Analgesic effects	MAF	—	Dar et al., 2005; Garrido-Suárez et al. (2014); Garrido-Suárez et al. (2020); Jaishree et al. (2009)
	SWM	—	Liu et al. (2016)
	Gent	—	—
Antipyretic effects	MAF derivatives **(53**~**58)**	Synthesized base on MAF	Kant et al. (2011)
Anti-tumor effects	MAF	—	Deng et al. (2018); Takeda et al., 2016; Xiao et al. (2020); Tang et al. (2019)
	SWM	—	—
Antibacterial effects	Aqueous extract of MR	Whole plant	Wang et al. (2018)
	Ethanol extracts of MR	Whole plant	Fang et al. (2018)
Hepatoprotective effects	MAF	—	Das et al. (2012); Bhattacharyya et al. (2014); Wu et al. (2017)
	SWM	—	—
Cardioprotective effects	MAF	—	Jiang et al. (2020); Liu et al. (2019)
Hypoglycemic effects	MAF derivatives **(59**~**61)**	—	Li et al. (2013)
	SWM	—	Vaidya et al. (2013b); Jaishree and Narsimha (2020)
Immunomodulatory effects	MAF	—	Rajendran et al. (2013)

### Anti-Inflammatory Effects

Anti-inflammatory effects contribute to heat-clearing and removing the phlegm, and purging fire and removing toxicity of MR. Anti-inflammatory effects are closely related to the suppressions of inflammatory cytokines and mediators such as TNF-α, IL-1β, IL-6, NO, PGE2 and others. Studies have demonstrated that compounds from MR exhibit anti-inflammatory properties.

A recent study has revealed that MAF inhibits mastitis induced by LPS (Qu et al., 2017). Briefly, 60 lactating female BALB/c mice were divided into six groups, including a control group, a LPS group, three MAF + LPS groups (5, 10, 20 mg/kg, 1 h prior to LPS treatment) and a MAF + LPS (20 mg/kg, 1 h post LPS treatment) group. After anesthesia, the teat duct was injected with 50 μl of LPS (0.2 μg/μl) followed by an intraperitoneal injection of MAF. 24 h after LPS injection, the animals were sacrificed and the mammary glands were collected for use. The results showed that MAF significantly attenuated LPS-induced histopathological injuries in mammary glands. Meanwhile, MAF markedly reduced increased MPO as well as the inflammatory cytokines NF-α, IL-1β, and IL-6. Further, MAF remarkably inhibit LPS-induced activation of NLRP3 inflammasome and NF-ĸB. These findings suggested that MAF alleviated LPS-induced mastitis by inhibiting NF-ĸB and NLRP3 signaling pathways. In this work, some issues are conspicuous 1) for only one dose of MAF (20 mg/kg) were used, it was far from efficient to evaluate the dose-efficacy relationship of this reagent; 2) the positive control was absent in this study; 3) NLRP3 inflammasome or NF-ĸB, which one is the main action pathway in MAF treating LPS-induced mastitis in mice? Actually, change in pharmacodynamics of MAF in this mastitis model needs to be evaluated in the presence of specific antagonist of NLRP3 inflammasome or NF-ĸB.

Additionally, MAF showed a good therapeutic effect on DSS-induced colitis in mice (Somani et al., 2016). 36 female BALB/c mice were allocated to three groups including a DSS-induced colitis group and two MAF-treated groups (30 and 60 mg/kg) (12 animals/group). The colitis model was established by successive administration of 5% DSS for 11 days. The animals in the treated groups were given MAF from day 5 to day 14 and once a day. On day 14, all the animals were sacrificed and colon specimens were collected for histopathological analysis and biochemical measurements. The results demonstrated that MAF (60 mg/kg) significantly attenuated reduced body weight and decreased the DAI score. MAF (60 mg/kg) markedly elevated decreased levels of CAT, GSH, and SOD, reduced MDA content and MPO activity. Further, MAF (60 mg/kg) remarkably reduced the colonic pro-inflammatory mediators TNF-α, IL-1β levels, and MMP-9 activity. Importantly, it significantly improved DDS-induced histopathological injuries of the colon. Molecular docking assay revealed that TNF-α and MMP-9 might be potential binding sites of MAF by using the GLIDE software. The limitations in the present study included 1) positive control drug was absent in this study, and sulfasalazine might be a better choice; 2) although the molecular docking assay suggested potential bindings of MAF and TNF-α and MMP-9, it is needed validation experiments such as affinity measurement by using a biosensor *in vitro*.

Recently, it has been documented that MAF displays a neuroprotective effect on early brain injury after SAH (Wang et al., 2017a). A SAH model was established by using endovascular perforation. A published grading scale was used to assess the severity of the SAH model in rats. SD rats were randomly allocated to five groups, including a sham group (n = 24), a SAH group (n = 36), a SAH + vehicle group (n = 36), and two SAH + MAF groups (20 and 100 mg/kg) (n = 36). The results showed that MAF (100 mg/kg) significantly reduced the mortality rate of the SAH rats, and restored neurological functions and decreased brain edema. MAF also alleviated oxidative stress injury in the SAH model. Furthermore, it decreased apoptosis of cortical cells by regulating mitochondria apoptosis-related proteins. In addition, MAF inhibited activation of NLRP3 inflammasome and NF-κB as well as inflammatory cytokines including TNF-α and IL-1β. Moreover, MAF remarkedly upregulated the expressions of Nrf2 and HO-1. Similar to the studies of Qu et al. (2017) and Somani et al. (2016), the positive control drug was absent in the present study and acivicin might be a better choice (Mendiola et al., 2020). ML385, a specific blocker for Nrf2 is encouraged to observe the pharmacodynamic change in MAF to further validate the relationship between Nrf2/HO-1 pathway and MAF.

It has been reported that swertiamarin (SWM) exhibit anti-inflammatory effects *in vitro* and *in vivo* (Saravanan, et al., 2014a; Saravanan et al., 2014b). *In vitro*, the anti-inflammatory effects of SMT were evaluated in IL-1β stimulated fibroblast-like synoviocytes (FLS) derived from AA rats by determining mRNA and protein expression levels of inflammatory and osteoclastogenesis mediators (Saravanan et al., 2014a). The results showed that SWM (10~50 μg/ml) significantly inhibited cell proliferation and NO production in a dose-dependent manner. It downregulated caspase 3, TNF-α, IL-6, PGE2, COX-2, iNOS, MMPs, and RANKL at both mRNA and protein levels. Further, the SWM treatment significantly downregulated the levels of p38 MAPKα in a concentration-dependent manner.

*In vivo*, chronic arthritis was induced by an injection of 0.1 ml of Freund’s complete adjuvant containing 1 mg of heat killed *mycobacterium tuberculosis* into right hind paw of a SD female rat (150~175 g) (Saravanan et al., 2014b). The animals were randomly divided into six groups (n = 6), including a normal group, a model group, a model plus vehicle group, an indomethacin group (2 mg/kg), and three SWM groups (2, 5, 10 mg/kg). 14 days post the adjuvant injections, the rats in the groups received various administrations for 14 days successively. The results suggested that SWM (2, 5, and 10 mg/kg) significantly inhibited the adjuvant paw edema in a dose-dependent manner. SWM markedly reduced the releases of proinflammatory cytokines including IL-1, TNF, IL-6 and proangiogenic enzymes such as MMPs, iNOS, PGE2, PPARc, and COX-2. In addition, it significantly increased the protein levels of anti-inflammatory cytokines like IL-10, IL-4. Furthermore, it also remarkably downregulated the proteins levels of NF-κB p65, p-IκB, p-JAK2 and p-STAT3 in both experimental animals and LPS-stimulated cells. More importantly, SWM attenuated bone destruction in the chronic arthritis rat model. These findings demonstrated that SWM might be a good anti-rheumatic agent. Actually, some aspects are encouraged to improve in this study. Lewis rat not SD rat is suitable to prepare this arthritis model. For the success ratio of this animal model isn’t 100%, it is necessary to confirm the successful establishment of this model before the groupings and administrations. Moreover, it is not appropriate to use indomethacin as the positive control in this study for it is not a specific blocker for the NF-κB/IκB or JAK2/STAT3 pathway.

Currently, the anti-inflammatory effects of gentiopicroside (Gent) have been investigated *in vitro* and *in vivo* (Wang et al., 2019b). *In vitro*, primary BMMs or PEMs were stimulated with LPS and interferon (IFN)-γ and then treated with Gent. *In vivo*, the anti-sepsis effect of Gent was evaluated in a mouse model challenged with LPS (40 mg/kg) injection. The findings suggested that Gent (1 mg/ml) downregulated inflammatory cytokines released from LPS/IFN-γ-induced BMMs and attenuated phosphorylations of IKKα/β and p65. *In vivo*, Gent (50 mg/kg) significantly elevated survival rate of the sepsis mice as compared with the control. Further, it downregulated the serum IL-1β and IL-6 protein levels, IL-1β, IL-6, and TNF-α mRNA expressions and M1 macrophage infiltration in the lung. In the cytotoxicity experiment, it is incomplete to test only a single concentration of this reagent. Also, the positive control drug was absent in the tests *in vitro* and *in vivo*.

### Antinociceptive Effects

Anti-inflammatory effects of MAF are closely associated with its anti-nociceptive effects. The analgesic effects are at least in part due to the inhibition of the NF-κB pathway activation (Piao et al., 2020). Once this pathway is activated, some mediators and enzymes such as iNOS and COX-2 are generated and involved in inflammation, pain, and oxidative stress (Rocha et al., 2020; Singh, et al., 2020).

Previously, a study showed that MAF and its derivatives displayed significant anti-nociceptive and antioxidant properties (Dar et al., 2005). MAF exhibited strong antioxidant activity (EC_50_ = 5.80 ± 0.96 μg/ml) by using DPPH assay, while acetyl (**44**) (EC_50_ = 14.20 ± 1.00 μg/ml) and cinnamoyl (**47**) (EC_50_ = 13.50 ± 1.78 μg/ml) derivatives possessed reduced activities as compared with MAF. Both the methyl (**45**) and its acetyl derivative (**46**) were inactive. Spectral studies showed that compared with H-5 and H-8, deuterium/hydrogen exchange at C-4 of the MAF derivatives cinnamoyl and the methyl led to reduced peak intensity of H-4 in CF_3_CO_2_D (Faizi et al., 2006). It resulted in less antioxidant activity of these compounds (**44**~**47**), which was consistent with the study of Dar et al. (2005). Based on these findings, it suggests that free hydroxyl groups and catechol moiety are essential for antioxidant activity. In addition, the acetic acid induced writhing test and hot plate test were used to evaluate the analgesic activity of MAF *in vivo*. MAF (42.2 mg/kg) significantly inhibited acetic acid-induced writhing in mice and its ED_50_ was slight lower than that of aspirin (12.53 ± 3.0 mg/kg vs. 18.20 ± 2.0 mg/kg). However, in the presence of naloxone, the inhibition of writhings was reduced to about 19% in MAF-treated group, suggesting that an opioid way was involved in MAF attenuating acetic acid induced pain. Unexpectedly, MAF prolonged retention time (RT) on the hot plate compared to the control at 90 min (14.0 ± 4.89 s vs. 5.50 ± 1.99 s). Moreover, the RT in the MAF group was also remarkably reduced and not significantly different from the control group in the presence of naloxone, which suggested a significant involvement of the opioid way.

The anti-hypernociceptive effect of MAF was also confirmed in a persistent and neuropathic pain model in rats (Garrido-Suárez et al., 2014). Acute spontaneous nociceptive behavior was evaluated by using a formalin test. Before the test, the animals were respectively treated with naloxone (0.1~1 mg/kg, i. p.), yohimbine (0.1~1 mg/kg, i. p.), methysergide (1~5 mg/kg, i. p.), L-NMMA (3–30 mg/kg or vehicle, i. p.) or an intrathecal injection of yohimbine (3~10 μg/20 μl). 15 min post the treatments, the animals received a dose of MAF (100 mg/kg, i. p.) or vehicle 20 min before the formalin injection. Morphine (5 mg/kg), clonidine (0.1 mg/kg) and DOI (1 mg/kg) were respectively selected as positive controls. After that, a CCI model was established in rat by ligating the common sciatic nerve on the left side under an anesthetized condition. The animals were then divided into three groups including a sham group, a CCI group, a CCI + MAF group (50 mg/kg). The animals were administered for 7 days (4 days prior to surgery and 3 days post CCI). Then a modification of the pin prick method was used to evaluate mechanical hypernociception of the hind paw. Behavioral responses to the pin prick were rated in accordance with the response of the paw. At the end of the experiment, the animals were sacrificed and the sciatic nerves were collected and stored in 10% formalin for H&E staining. After that, pathological changes in the nerve sections were observed under a light microscope. In addition, the MTT method was used to assess the protective effect of MAF on glutamate-mediated injury in PC12 cells.

The results showed that MAF (50, and 100 mg/kg) had no significant effect on phase I nociceptive activity as compared with vehicle controls, while the cumulative mean of licking/biting times (s/5 min) exclusivity during phase II in the MAF (50, 100 mg/kg) groups was lower than that in the vehicle control (VC) group (MAF50: 75 ± 25 vs. MAF100: 232 ± 19 vs. VC: 556 ± 32, *p* < 0.001). The pretreatment of yohimbine (1 mg/kg) significantly reversed the anti-hypernociceptive effects of clonidine (0.1 mg/kg) and MAF (100 mg/kg) during phase II of the formalin test. Naloxone (1 mg/kg) partially reversed the effect of MAF as compared with morphine (5 mg/kg) in the phase II. The pretreatment of methysergide (5 mg/kg) remarkably reversed the effect of DOI (1 mg/kg) compared to the controls, but did not influence the effect of MAF during the phase II. Compared with the VC, high-dose L-NMMA (30 mg/kg) significantly reduced the cumulative mean of licking/biting during phase II (L-NMMA: 210 ± 32 vs. MAF100: 139 ± 41 vs. VC: 526 ± 50, *p* < 0.001). The L-NMMA pretreatment (30 mg/kg) markedly enhanced the effect of MAF. The pretreatment of nitric oxide precursor L-arginine reversed reduced licking/biting response of MAF in this phase. In addition, low doses of ketamine (0.3, and 1 mg/kg) had no influences on licking/biting behavior in the tonic phase. However, its administration at 1 mg/kg significantly enhanced the effect of MAF (100 mg/kg). Further, the pretreatment of intrathecal yohimbine (10 μg/20 μl) significantly reversed the effect of MAF in the phase II. L-NMMA (20 μg/20 μl) didn’t change the effect of MAF. It indicated that the NOS inhibitor significantly enhanced the anti-hypernociceptive effect of MAF. Meanwhile, the L-NMMA pretreatment minimized the cumulative mean of licking/biting behavior. The pathological analysis demonstrated that MAF significantly attenuated CCI-induced Wallerian degeneration-related changes in the nerves. The MTT assay revealed that L-glutamate inhibited the PC12 cells by approximately 48% under a concentration of 50 mM of MAF. The MAF pretreatment (100 and 250 μg/ml) significantly elevated the cellular viability from 61 to 71%. In summary, it suggested that MAF exhibited its anti-hypernociceptive activity in a tonic pain model. The antinociceptive activity of this compound was mainly mediated by spinal α_2_ adrenergic receptors at least in part in cooperation with the opioid system. This study systemically investigated anti-hypernociceptive effect of MAF and its possible mechanisms of action. The experiment was designed completely but not complicatedly, which was efficient to illuminate the efficacy and mechanism of MAF. Some minor issues need improvements including applications of gene knock-out animals *in vivo*.

Recently, Garrido-Suárez et al. (2020) proposed therapeutic potential of MAF as a multi-targeted compound in mixed OA pain based on three main aspects including the preclinical evidence of this xanthone, applications of formulas containing MAF in some preliminary clinical studies on musculoskeletal or neuropathic pain, and speculations regarding its possible mechanism of action in accordance with research advances in OA pain.

Also, the anti-nociceptive effects of SWM were evaluated by the hot plate, tail immersion, and acetic acid-induced writhing (Jaishree et al., 2009). These three different sets of mice were randomly divided into four groups, including a control group, a paracetamol group (100 mg/kg), and two SWM groups (100, and 200 mg/kg) (n = 6). All the animals were received treatments 30 min prior to these tests. The results showed that SWM (100, and 200 mg/kg) significantly increased the latency period after 30 and 45 min as compared with the control. The SWM treatment markedly elevated the tail withdrawal reflex at doses of 100 and 200 mg/kg. In addition, the SWM pretreatment (100, and 200 mg/kg) remarkably reduced the number of writhing with protection rates of 51.43 and 56.08%, respectively. It suggested that SWM exhibited strong anti-nociceptive effects on both peripheral and central pains. However, paracetamol was inappropriately used as the positive control in the hot plate and tail immersion tests, and opioids were rational choices.

Currently, anti-nociceptive effects of Gent were evaluated in a chronic constriction injury-induced neuropathic pain model in mice (Liu et al., 2016). The CCI model was induced in accordance with the method described previously. 60 male mice weighing 18–22 g were divided into six groups including sham + NS; CCI + NS; CCI + Gent (25, 50, 100 mg/kg); and CCI + pregabalin (10 mg/kg) (n = 10). 7 days after the surgery, the animals in the groups were treated for 7 days successively and once a day. Recorded and evaluated behavioral parameters and SFI from day 0, 7, 8, 10, 12, and 14 after the surgery. After the last behavioral test, the mice in the groups received electrophysiological examinations. In addition, the rotarod test and spontaneous locomotor activity tests were performed in another 40 mice. The animals were divided into four groups as well as the parameter/observation and SFI experiments but without sham + NS and CCI + pregabalin. 15 min post the treatments, the motor coordination and spontaneous locomotor activity tests were carried out from day 14 after the CCI surgery. 7 day after the surgery, significant mechanical allodynia was observed in the CCI mice. Gent (25~100 mg/kg) markedly elevated decreased PWT in a dose-dependent manner as compared with the CCI + NS group. Compared with the model group, the Gent treatments (25~100 mg/kg) significantly reduced rising on the counts of PWT caused by cold allodynia in the CCI mice 10, 12, and 14 days after the surgery. In addition, Gent (50, and 100 mg/kg) reversed of the reduced PWL as compared with the model group. Furthermore, Gent (50, and 100 mg/kg) restored sciatic nerve function and sensory nerve action potential amplitudes. However, Gent (50, and 100 mg/kg) had no significant influence on motor coordination and spontaneous locomotor (exploratory) activity.

### Antipyretic Effects

Antipyretic effects are based on heat-clearing and removing toxicity of MR. The antipyretic effects are associated with amelioration of inflammatory actions of cytokines.

A study has revealed that MAF and its novel analogues displays significant antipyretic effects in a yeast-induced pyrexia mouse model (Kant et al., 2011). Briefly, 80 Swiss albino mice were randomly divided into 16 groups (n = 5) including a control group, an ASA group (150 mg/kg), MAF (100, and 200 mg/kg) groups, and its six analogues groups (100, and 200 mg/kg for each analogue) groups. Aqueous suspension of yeast was subcutaneously injected into the nape of the neck of the mice (20 ml/kg). Subsequently, the animals received various treatments. The rectal temperature of each mice was recorded 0, 1, 2, and 3 h post the administrations. The results indicated that ASA, MAF and its analogues compounds **(55)**, **(57)**, and **(58)** exhibited significant reductions in rectal temperature within the whole observation period (3 h), whereas compounds **(53)**, **(54)**, and **(56)** showed no antipyretic effects at 100 mg/kg at 1 h after the administrations. Although antipyretic effects of MAF and its six analogues have been displayed in the present study, the SARs of these compounds still remain unknown. Furthermore, mechanisms for the antipyretic effects of MAF and its analogues are needed for further investigations.

### Anti-tumor Effects

A study has reported inhibition effects of MAF on migration and invasion of breast cancer cells (Deng et al., 2018). The MTT method was used to assay viability of MDA-MB-231 and MCF-7 cells. It demonstrated that the IC_50_ value for MAF was 10 μM for the MDA-MB-231 cells. Western blot analysis showed that MAF significantly downregulated protein levels of Rac1/Cdc42, phospho-Rac1/Cdc42, WAVE2, Arp2, and Arp3 in the MDA-MB-231 cells as compared with the controls. The immunofluorescent staining assay suggested that MAF (10 μM) markedly reduced positive WAVE2 staining in the MDA-MB-231 cells compared with the controls. In addition, MAF (10 μM) significantly inhibited cell migration and invasion of the breast cancer cell by approximately 62 and 65%, respectively. The present study indicated that MAF inhibited the migration and invasion of the MDA-MB-231 cells, which might be associated with the suppression of the Rac1/WAVE2 signaling. This is a preliminary study to evaluate the efficacy and mechanism of MAF in inhibiting migration and invasion of breast cancer cells *in vitro*. Actually, the specific agonist or antagonist of Rac1/WAVE2 pathway is needed to investigate the pharmacodynamics changes to clarify the mechanism of this xanthone. In addition, the concentration of MAF used in these test was equal to its IC_50_ value (both 10 μM), which resulted in cytotoxicity.

Recently, anti-metastatic melanoma effect of MAF *in vivo* has been documented (Takeda et al., 2016). Briefly, in spontaneous pulmonary metastasis studies, 50 μl of B16BL6 cells (1 × 10^5^ cells) were injected into footpad of each 6-week-old male C57BL/6 mouse. Then the animals were randomly allocated to four groups including a control group (0.1% DMSO) and three MAF groups (50, 100, 200 mg/kg). In addition, the mice in the shame control were only treated with MAF throughout experimental period. 21 days after the injections, the footpad tumors were surgically removed. After that, the mice were respectively administrated with 0.1% DMSO in the tumor control group and MAF (50, 100, and 200 mg/kg) in the MAF groups once a day. The animals were successively received the treatments for another 21 days and then sacrificed. The metastasis nodules in the lungs were observed and counted. For subcutaneous tumor growth studies, the experimental design was similar to the pulmonary metastasis studies except that the tumor growth was measured every day from day 8 to 17. The footpad tumor was surgically excised at 17 days and stored on dry ice for western blot, qRT-PCR, and collagenase activity assays. For survival studies, 0.2 ml of B16BL6 cells (1 × 10^5^ cells) were injected into each mouse *via* tail veins. Next, the total of 60 mice were separated into four groups (n = 15), including a tumor control group receiving 0.1% DMSO and three other groups administered with MAF (50, 100, and 200 mg/kg) once a day from the inoculation. The deaths of the animals were recorded and the survival curves were drawn. The results showed that MAF reduced the number of lung metastatic nodules as compared with the tumor control. Meanwhile, MAF (50, 100, and 200 mg/kg) remarkably reduced the volume of footpad tumor in a dose-dependent manner. Furthermore, MAF downregulated expressions of NF-κB and phosphorylated NIK, IKK, and IκB, and upregulated expression of IκB, whereas it had no significant effect on phosphorylations of ERK1/2, Akt, mTOR, and p38. The results above suggested that MAF suppressed metastatic melanoma *in vivo* by selectively inhibiting the NF-κB pathway *via* suppressing the NIK activation. There are some issues including absence in the MAF dose used in this control, positive control drug, and specific antagonist of NF-κB.

Recently, it has revealed that SWM suppresses proliferation, migration, and invasion of HCCs (Xiao et al., 2020). The cell viability was assayed by the CCK8 method. Cell proliferation was detected in a plate cloning experiment. Cell migration and invasion was detected using trans-well assay and the RTCA system. Differential genes in HepG2 cells treated with and without SWM were analyzed by microarray. *In vivo*, a nude mouse model was used to assess the anti-tumor activity of SWM. SWM significantly suppressed cancer cell proliferation in a concentration-dependent manner. The IC_50_ values of SWM were respectively 87.96 ± 1.41, and 56.49 ± 0.76 μg/ml for HepG2 and Huh7. SWM significantly reduced the number and size of cellular colonies of the HepG2 and Huh7 under concentrations of 70 and 50 μg/ml, respectively. SWM remarkably inhibited the cell proliferation, migration, and invasion of the HepG2 and Huh7. *In vivo*, intratumoral injections of 10 μg of SWM markedly decreased the volumes and weights of the tumors in the nude mice. Microarray analysis showed that FRAT1 was downregulated in both SWM-treated HepG2 and Huh7. Transfection with FRAT1 lentiviral vector (FRAT1-OE) not only promoted cell proliferation, migration, and invasion, but also attenuated antitumor effect of SWM. The SWM treatment significantly upregulated levels of p-β-catenin and GSK-3β, and it downregulated FRAT1, β-catenin, and Cyclin D1. Furthermore, inhibition effect of the SWM treatment on the Wnt/β-catenin pathway was reduced after the transfection of FRAT1-OE. The results above suggest that SWM exerts its anti-tumor effects mainly *via* inactivating the FRAT1/Wnt/β-catenin signaling axis. In the present study, it is far from enough to reflect the dose-efficacy relationship for only a single dose of SWM was used in the *in vivo* experiment. Furthermore, the positive control drug was absent in this part experiment.

Subsequently, Tang et al. (2019) reported the differentially expressed genes in HCCs after treated with SWM by bioinformatics analysis. The results demonstrated that SWM significantly attenuated the viability and invasion and elevated the apoptosis of HepG2 cells. *In vivo* the growths of the xenograft in the nude mice were significantly inhibited after the SWM treatment. The microarray analysis indicated that PI3k-Akt was the most significantly regulated pathway in the HepG2 cells after treated with SWM. Chemical genomics-based virtual screening and K Nearest Neighbor with K = 3 (3NN) respectively predicted 47 and 21 targets of SWM. Among them, two targets were predicted to be prominent targets and then validated as JUN and STAT3. Although the microarray analysis predicted the differential expressed genes exposed to SWM, the outcomes needconfirmation by qRT-PCR and western blot techniques.

### Antibacterial Effects

Wang et al. (2018) evaluated the therapeutic effects of MR decoction (MRD) on pneumonia caused by *Streptococcus pneumoniae* in mice. 30 thirty clean KM mice were randomly divided into three groups, a control group, a model group, and a MRD-treated group (n = 10). The *Streptococcus pneumoniae* induced pneumonia mouse model was established by an intranasal instillation of 100 μl of bacterial suspension (3 × 10^7^ CFU/ml). The mice in the MRD-treated group were administrated with MRD decoction (4 g/kg) every 8 h. When eye closure and tachypnea occurred, the animals were sacrificed and blood samples were collected. ELISA was used to determine serum CRP, PCT, IL-6, and IL-8 levels. The pathological changes in the lungs were observed under an optical microscope. The results indicated that MRD significantly lowered serum CRP, PCT, IL-6, and IL-8 levels as compared with the control. Importantly, MRD markedly improved damaged histopathology of the lungs, including reduced inflammatory secretion and inflammatory cellular infiltration. However, as well as the study of Li et al. (2019), only single dose of this preparation was used and the positive control drug was absent. For the successive rate of this modelling is not 100%, it is necessary to provide the standard for the successful establishment of this model before groupings and administrations. Further, the active components in MRD need to be clarified by HPLC or other chemical analyses. Recently, the anti-bacterial activity of MR extracts were determined in *Escherichia coli*, *Bacillus subtilis*, and *Staphylococcus aureus* by using the Disc diffusion test (K-B method) (Fang et al., 2018). The results showed that the order of antibacterial ability of the extracts from MR was tissue culture plantlet root > whole grass > ground part; the order of the inhibitory effects of the whole grass and the tissue culture plantlet root was *Bacillus subtilis* > *Escherichia coli* > *Staphylococcus aureus*; the order of the inhibitory effect of ground part was *Escherichia coli* > *Bacillus subtilis* > *Staphylococcus aureus*. Bacteriostatic ring diameters of the culture plantlet root, whole grass, and ground part were respectively (11.33 ± 0.44), (9.42 ± 0.45), (8.34 ± 0.42) mm against *Escherichia coli*, (11.67 ± 0.38), (9.05 ± 0.72), and (6.00 ± 0.00) mm against *Bacillus subtilis*, and (9.67 ± 0.53), (8.36 ± 0.44), and (9.33 ± 0.44) mm against *Staphylococcus aureus* when the concentrations of extracts reached 0.125 g/ml. These findings suggested that the culture plantlet root could be developed into natural bacteriostatic agents. Actually, the detailed MIC values for these extracts against the three bacteria were not provided directly. Further, the concentration of 0.125 g/ml used in this study was high for these extracts to exert their antibacterial effects.

Additionally, SWM and sweroside **(4)** exhibit anti-bacterial activities (Kumarasamy et al., 2003). Both of these two compounds inhibited the growth of *Escherichia coli, Bacillus cereus, Citrobacter freundii,* and *Bacillus subtilis*. Further, SWM significantly inhibited the growth of *Serratia marcescens* and *Proteus mirabilis*, as well as sweroside against *Staphylococcus epidermidis*. The brine shrimp lethality bioassay revealed that SWM and sweroside showed general toxicities with LC_50_ values of 8.0 and 34.0 μg/ml, respectively.

### Hepatoprotective Effects

Dar et al. (2005) evaluated the hepatoprotective activity of MAF by using a 20% CCl_4_ (1.5 ml/kg) induced liver injury model in rat. The results demonstrated that the serum ALT and AST levels in the CCl_4_ group were significantly higher than those in the control group. The MAF treatments (0.1, 1, 10 mg/kg) markedly reduced increased serum ALT and AST levels, which further supporting the free radical scavenging property in the *in vivo* animal model.

In addition, it has been documented that MAF exhibits hepatoprotective activity in a D-galactosamine (D-GAL) induced acute liver injury model in rat (Das et al., 2012). It demonstrated that the activities of serum ALP and ALT and the levels of triglycerides, total cholesterol, lipid-peroxidation were elevated, while the levels of serum total proteins, albumin and cellular GSH were reduced after the D-GAL challenge. *In vitro*, the D-GAL treatment (5 mM) resulted in apoptosis and necrosis and increased ROS and NO production in hepatocytes. Also, D-GAL markedly enhanced the nuclear translocation of NF-κB and increased iNOS protein expression. Similarly, it also elevated TNF-α, IFN-γ, IL-1β, IL-6, IL-12, IL-18 and decreased IL-10 mRNA levels as well as Nrf2 protein expression. However, MAF significantly reversed the D-GAL-induced adverse effects all above. In the present study, it was difficult to reflect the dose-efficacy relationship of MAF for a single dose of this reagent was used. In addition, the positive control drug and the specific antagonists of Nrf2/NF-κB pathways were absent.

The major disadvantage of MAF is reduced biological activity for its poor absorption, low bioavailability and rapid elimination of after administration. Bhattacharyya et al. (2014) prepared a phospholipid complex of MAF to overcom these limitations and then evaluated its hepatoprotective activity and bioavailability in a CCl_4_-induced liver injury rat model. The results showed that compared with pure MAF, the complex has a significantly enhanced hepatoprotective effect at the same dose level (30 and 60 mg/kg). In addition, the complex reduced increased levels of serum hepatic marker enzymes like GOT and GPT, ALP, Tbil, and total protein and liver antioxidant enzymes such as GSH, GPx, GST, GRD, SOD, CAT, and TBARS in CCl_4_-treated rats’ plasma. Also, the complex remarkably increased the bioavailability of MAF in rat serum as compared with that of pure MAF at the same dose. Further, the elimination t_1/2_ was prolonged from 1.71 ± 0.12 h to 3.52 ± 0.27 h. This study provided a good reference to utilize MAF to protect damaged hepatic function in clinic.

Also, SWM exhibits a hepatoprotective effect on CCl_4_-induced hepatic damage (Wu et al., 2017). The results demonstrated that SWM significantly attenuated increased levels of serum ALT, AST, and ALP and histopathological injuries in the livers. It also reduced MDA content and elevated activities of SOD, GPx, and GSH. Meanwhile, SWM markedly downregulated increased levels of inflammatory cytokines/chemokines such as iNOS and IL-1β. In addition, the protein expressions of CYPs, efflux transporters and PDZK1 in the SWM groups were higher than those in the CCl_4_ group. Furthermore, SWM significantly increased the protein expressions of Nrf2, HO-1, and NQO1 as compared with the CCl_4_ group. Similar to the study of Das et al. (2012), the positive control drug and the specific antagonists of Nrf2/HO-1 pathway were absent.

### Cardioprotective Effects

Currently, it has been reported that MAF have cardioprotective and anti-apoptotic effects in heart failure induced rats (Jiang et al., 2020). MAF significantly improved damaged cardiac functions. The pathology assessment showed MAF reduced inflammatory infiltrations, broken myocardial fibers, and apoptosis in cardiomyocytes. Further, it downregulated Caspase-3 and Bax and upregulated Bcl-2. Actually, it may be more reasonable to allocate the animals after validating the successful establishment of this ISO-induced heart failure model by ECG.

In a coronary artery occlusion-induced myocardial ischemia-reperfusion injury (I/R) model in rats, MAF exerted a cardioprotective effect *in vitro* and *in vivo* (Liu et al., 2019). *In vitro*, MAF improved H9c2 cell activity induced by hypoxia/reoxygenation- (H/R-). In addition, in the H/R-induced H9c2 cells, MAF remarkably reduced oxidative stress and protein expressions of inflammatory pathway. *In vivo*, MAF (50 mg/kg) markedly reduce myocardial injury by TTC staining analysis. Further, it attenuated myocardial oxidative stress and proinflammatory cytokines in the IR rats. In this study, the specific antagonists of MAPK/Nrf-2/HO-1/NF-κB pathway was absent and only a single dose of MAF was used.

### Hypoglycemic Effects

A STZ-induced hyperglycemic mouse model was used to evaluate the hypoglycemic activity of three esterified-derivatives of MAF. Biopsy inspection was used to check the islet cells (Li et al., 2013). PAM **(59)** (0.25, 0.5 mmol/kg), HBM **(61)** (0.125, 0.25, 0.5 mmol/kg) remarkably reduced increased blood glucose levels in the hyperglycemia mice. MAF (0.5, 1 mmol/kg), PAM **(59)** (0.125 mmol/kg) and HPM **(60)** (0.125 mmol/kg) exhibited marginal hypoglycemic activity. The histological analysis demonstrated that PAM-, HPM-, and HBM-treated islet cells recovered from damage caused by the STZ, which was more significant than MAF-treated. These three esterified-derivatives of MAF had more potential hypoglycemic activity than MAF. The SAR analysis suggested that the larger esterification moieties (alternatively the higher lipid-solubility) the more potent hypoglycemic activity according to an order as follows: no ester, acetyl, propionyl, and butyryl). Thus, it is possible to transform MAF to strong anti-diabetes drug by esterification.

A study reported that gentianine, an active metabolite conferred SWM possessed anti-diabetic activity in 3T3-L1 cells, which was associated with upregulated PPAR-γ gene expression (Vaidya et al., 2013b). Dipocytes differentiation was assayed in 3T3-L1 cell culture. RT-PCR was used to determine the mRNA expressions of PPAR-γ and GLUT-4. The results showed that SWM had no significant effect on adipogenesis as well as the mRNAs of PPAR-g and GLUT-4. However, gentianine significantly increased the mRNA expressions of adiponectin and adipogenesis. This study preliminarily investigated the anti-diabetic effect of the metabolite of SWM, gentianine. The mechanism of action of this active metabolite is encouraged in the further study.

Recently, a study has reported that a combination of SWM and quercetin (CSQ) ameliorates hyperglycemia in a STZ-induced type two diabetes mellitus rat model (Jaishree and Narsimha, 2020). CSQ (50, 100 mg/kg) significantly reduced levels of LDL, triglycerides, total cholesterol and increased HDL level as compared with the control. Also, the CSQ treatment markedly upregulated decreased levels of serum GSH, SOD, catalase and GPx and downregulated increased lipid peroxide level. Further, CSQ significantly improved pathological injuries of pancreas by increasing pancreatic islets of Langerhans and vacuolization. However, the mass ratio of SWM and quercetin was not shown in the present study. Moreover, the positive control drug was also absent in the design of the experiment.

### Effects on Gastrointestinal Functions

It has been reported that Gent significantly attenuated gastrointestinal motility disorder (GMD) induced by stress (Ruan et al., 2015). Briefly, the SD rats were randomly divided into six groups including a control group, a model group, a mosapride group (2.5 mg/kg, positive control), three Gent groups (40, 80, and 160 mg/kg) groups (five male and five female rats in each group). The stress-induced GMD rat model was established in accordance with the reported reference (Hu et al., 2011). The results showed that Gent significantly increased gastric emptying and intestinal propelling. Meanwhile, it remarkably increased plasma GAS and reduced SST level. Further, the MTLR expressions were higher in gastric antrum, duodenum, jejunum and ileum than the model control after the Gent treatments. In addition, it lowered the VIPR2 expression in duodenum. This study illuminated the effects of Gent on gastrointestinal functions and provided a potential value of this compound in treating stress-induced GMD.

### Skin Protective Effects

Recently, Yang has evaluated anti-skin lesions property of *Gentiana scabra* Bunge roots in a contact dermatitis mouse model (Yang et al., 2019). The model was induced using DNFB as described previously (Yang et al., 2017). After the establishment of this model, ethanol extract of *G. scabra*, roots and rhizomes (EEGS) (6, 18, 60 µg/day in EAOO) and dexamethasone (DEX) (15 µg/day in EAOO) were respectively applied for 6 days from day 9–14. All the animals were sacrificed on day 15. The revealed that EEGS alleviated contact dermatitis-induced skin lesions in mice as compared with the model control. Further, EEGS prevented hyperkeratosis, epidermal hyperplasia and immune cell infiltration, which was associated with inhibitions of related cytokines including TNF-α, IFN-γ, IL-6, and MCP-1 in the inflamed tissues.

### Immunomodulatory Effects

Rajendran et al. (2013) found that MAF exhibited immunomodulatory effects in a benzo(a)pyrene-induced lung carcinogenesis animal model. 30 male Swiss albino mice weighing 23–26 g were randomly separated into six groups (n = 5): a control group (received corn oil as a vehicle), a model group (administrated with 50 mg/kg B(a)P from the 2nd to 6th week and twice a week), a MAF pre-treated group (100 mg/kg, treated from the 1st week to the 18th week, twice a week), a MAF post-treated group (100 mg/kg, treated from the 12th week to the 18th week, twice a week), a single MAF-treated group (100 mg/kg, treated for 18 weeks). At the end of the experiment, the blood samples were collected to count immunocompetent cells, determine immune function, and assay the NBT reduction. Levels of IgG, IgA, IgM, and soluble immune complex were detected in the coagulated blood samples. The findings demonstrated that MAF upregulated decreased phagocyte index, avidity index, and SIC levels, and NBT reduction induced by B(a)P. Further, MAF significantly elevated the levels of IgG and IgM and reduced the IgA level as compared with the B(a)P-treatment. Additionally, it remarkably reversed increased lipid peroxidation and decreased catalase and SOD activities in the lymphocytes, polymorphonuclear cells, and macrophages from B(a)P-treated mice. It suggested that MAF exerted the immunomodulatory effects *via* attenuating intermediate-induced oxidative stress in immune cells including lymphocytes, neutrophils, and macrophages.

## Toxicology

Until now, no studies have been documented on the toxicology of the extracts of MR. Actually, the toxicology of its characteristic compound MAF has been reported (Fan, et al., 2015; Wang et al., 2017b).

Fan et al. (2015) evaluated the acute toxicity of MAF in ICR mice (half male and half female) by using Horn’s method. 32 mice were divided into four groups (n = 8), including a control group and three MAF groups (4.64, 10, 21.5 g/kg). The animals were administrated once (0.2 ml/10 g) and then observed for consecutive 7 days. In addition, the genetic toxicity of MAF was assessed by using Ames test, mouse bone marrow micronucleus frequency test, and mouse sperm deformity test. The results showed that no death and no significant toxicity of MAF was observed in the mice. The LD_50_ value of MAF were larger than 21.5 g/kg, suggesting non-toxicity of this compound. There were no significant differences in PCE micronucleus frequency, ratio of PCE and NCE, and sperm abnormal rate among the negative control group and the MAF-treated groups. The Ames test demonstrated that MAF had no mutagenic effects on *Salmonella typhimurium* strains TA97, TA98, TA100, and TA102.

Also, the acute toxicity of MAF was examined in KM mice and Beagle dogs (Wang et al., 2017b). The mice and dogs were respectively divided into control groups and MAF-treated groups. The treated animals were administrated with the MTD of MAF (respectively 54 g/kg for mice and 9 g/kg for dogs) and then observed for consecutive 14 days. The results revealed that no death was found in the mice and dogs. No pathological changes were found in the organs after dissection. Grey feces were found once in dogs with an occurrence rate of 25%. In addition, no abnormality was found in biochemical indices. This study had some similarity to the study of Fan, while it seemed that MTD not LD_50_ was more appropriate to evaluate the acute toxicity of MAF.

In summary, these studies preliminarily evaluate the acute and reproductive toxicity of MAF. It is encouraged to assess its general, nervous, cardiovascular, respiratory toxicity more comprehensively in various experimental animals for referring clinical practice in the future.

## Clinical Uses

Nowadays, MR has been made into some preparations including Feilike Jiaonang, Kangfuling Jiaonang, Ernong Wan, Kudan Wan, and Longdanxiegan Wan in combination with other TCMs to treat cough, chronic bronchitis, asthma (Li and He, 2014; Chen et al., 2018), dysmenorrhea, appendagitis (Zheng et al., 2019), deafness, tinnitus, icteric hepatitis, acute and chronic hepatitis, hematuria due to gonorrheal infection, etc (Table 4), which is a prolongation of the traditional uses of this plant. Until now, no single extract or compound from MR has been applied in clinic to cure diseases in China. Therefore, it is necessary to study and develop potentially therapeutic extracts or compounds from MR in accordance with the previous studies.

**TABLE 4:** Clinical uses of MR in China.

Name of TCM patent formula	Components	Modern uses	Usage
Feilike jiaonang	*Scutellaria baicalensis* georgi 173 g, *Kitagawia praeruptora* (dunn) pimenov 167 g, *Stemona japonica* (blume) miq. 160 g, MR 150 g, *Firmiana simplex* (L.) W.Wight 130 g, *Scleromitrion diffusum* (willd.) R.J.Wang 120 g, *Aster ageratoides* turcz 96g. [For 1000 capsules (0.3 g/capsule)]	Curing cough, phlegm, asthma and chronic bronchitis	Take orally 3 times a day (0.9–1.2 g once)
Kangfuling jiaonang	*Persicaria perfoliata* (L.) H.Gross 600 g, *Sophora flavescens* aiton 300 g, *Phellodendron amurense* rupr. 150 g, *Spatholobus suberectus* dunn 300 g, *Leonurus japonicus* houtt. 300 g, MR 200 g, *Smilax glabra* roxb. 150 g, *Angelica sinensis* (oliv.) diels 180 g	Curing low menstrual flow, dysmenorrhea, appendagitis	Take orally 3 times a day (1.2 g once)
Ernong wan	MR 500 g, *Scutellaria baicalensis* georgi 500 g, *Rehmannia glutinosa* (gaertn.) DC. 500 g, *Alisma plantago-aquatica* subsp. Orientale (sam.) sam. 500 g, *Akebia quinata* (thunb. Ex houtt.) decne. 500 g, *Gardenia jasminoides* J. Ellis 500 g, *Angelica sinensis* (oliv.) diels 500 g, *Acorus calamus* var. angustatus besser 500 g, *Glycyrrhiza uralensis* fisch. Ex DC. 500 g, *Saigae Tataricae* cornu 25 g	Curing dizziness, headache, deafness, tinnitus	Take orally 2 times a day (7 g once)
Kudan wan	*Sophora flavescens* aiton 933, MR 666 g, *Phellodendron amurense* rupr. 266 g, *Rheum palmatum* L. 133 g, *Curcuma aromatica* Salisb.133 g, *artemisia capillaris* thunb. 2000 g, porcine bile paste 133 g, *Hyssopus officinalis* L. 133 g	Curing icteric hepatitis, acute and chronic hepatitis	Take orally 2–3 times a day (5–10 g once)
Longdanxiegan wan	MR 120 g, *Bupleurum chinense* DC. 120 g, *Scutellaria baicalensis* georgi 60 g, fried *Gardenia jasminoides* J.Ellis 60 g, *Alisma plantago-aquatica* subsp. Orientale (sam.) sam. 120 g, *Akebia quinata* (thunb. Ex houtt.) decne. 60 g, salted *Plantago asiatica* L. 60 g, alcohol *Angelica sinensis* (oliv.) diels 60 g, *Rehmannia glutinosa* (gaertn.) DC. 120 g, *Glycyrrhiza uralensis* fisch. Ex DC. 60 g	Curing dampness and heat in liver and gallbladder, dizziness, red eyes, tinnitus and deafness, ear swelling and pain, red-colored urine	Take orally 2 times a day (6–12 g once)

The full taxonomic names of the species have been validated using https://mpns.science.kew.org/mpns-portal/.

## Conclusion

In the present study, the traditional uses, phytochemistry, pharmacology toxicology and clinical uses of MR have been focused and reviewed comprehensively.

It reveals that MR, an ethnomedicine in southwest China, exhibits biological activity such as anti-inflammatory, antinociceptive, antipyretic, and other effects, which is associated with its traditional uses. The phytochemistry assays show that MAF is a characteristic substance of this plant. Few studies have reported the toxicology of the extracts from MR. Nowadays, in China some Chinese patent drugs such as Feilike and Kangfuling capsules have been marketed to treat lung-related and gynecological diseases. Although this review summarized the related status and development of various aspects of MR, some issues still need to be solved to utilize this plant better.

Although phytochemistry studies have isolated some compounds from the whole plant and roots from this plant, no study has documented the compounds separated from the fruits. Thus, the chemical studies on the fruits of this plant need strengthenment.

For MAF is a characteristic material in MR, it is usually used to detect the quality of this plant (Wu et al., 2010; Shen et al., 2016; Liu et al., 2020). These findings suggested that the content of MAF was unstable in different batches from a same production area, in different medicinal parts of this plant, from, or in different production areas (Wu et al., 2010; Shen et al., 2016), which proposed a challenge for medicinal applications of this plant in clinic. In view of this, quality standards are necessary to be established for medicinal components from MR. Further, quality of preparations or drugs from this medicinal plant will be ensured by establishing good agricultural practice (GAP) bases for this plant.

It has been mentioned that the pharmacological studies are mainly focused on the characteristic compounds such as MAF, SWM, and Gent of MR. However, the pharmacological reports on the extracts from this plant are few mainly due to the lacks in the understanding of the chemical components in the extracts. So, the chemical study on MR needs to be strengthened to better conduct the biological test of these extracts.

The experimental designs for some pharmacological studies on the extracts and characteristic compounds are incomplete for example 1) the absence of positive control drugs results in lacks in comparability; 2) absence of signal pathway blockers results in incomplete conclusions for mechanisms of action; 3) only a single dose of the tested drug used causes the lack in reflecting the dose-efficacy relationship.

Further, the SAR analyses MAF and its derivatives are encouraged in the further studies. In the antioxidant activity test, the free hydroxyl groups and catechol moiety are essential for antioxidant activity of MAF. In the antipyretic analysis, no analogues are better than MAF. Moreover, the SARs of these compounds still remain unknown. In the hypoglycemic activity test, those esterified-derivatives had more potential hypoglycemic activity than origin compound MAF. The SAR analysis suggests that the larger esterification moieties (alternatively the higher lipid-solubility) promotes the more potent hypoglycemic activity. Thus, it is necessary to further assay the SAR of MAF and its derivatives.

In addition, anti-inflammatory properties of MAF, SWM, and Gent contribute greatly to other effects of these characteristic compounds. These three compounds mainly exert their anti-inflammatory effects by inhibiting inflammation related signalling pathways such as JAK2/STAT3/NF-ĸB and downstream inflammatory mediators such as TNF-α, IL-1β, IL-6, MMPs, iNOS, and PGE2.

Until now, except for MAF, few toxicological studies have been conducted in extracts or compounds of MR. Therefore, studies on acute toxicity, chronic toxicity, safety pharmacology, and others toxicity should be performed to comprehensively evaluate toxicities of extracts or compounds from this plant in various experimental animals. In addition, the toxicological studies on MAF are still preliminary and also need to be strengthened.

The Chinese patent drugs containing MR marketed in local or overseas are few. Thus, it is necessary to develop other TCM preparations containing MR guided by the chemical analyses and pharmacological tests.

Except lung-related and gynecological diseases, pain caused by inflammation or nervous pathology, tumor, and diabetes mellitus may enter the therapeutic scopes of this plant based on the reviewing the pharmacological research progress on MR.

Finally, the pharmacokinetics of the extracts or compounds from MR are encouraged in random, double-blinded, multi-center clinical trials for important references for clinical applications of this plant.

Taken together, the present study provides a critical and comprehensive analysis on traditional uses, phytochemistry, pharmacology, toxicology, and clinical applications of MR. Also, some limitations in the research and development of this plant are raised and solved in this review. Based on this, we hope to highlight the potential value of MR and provide some new research directions and cues for this medicinal plant in southwest China.

## Glossary

AA: adjuvant induced arthritis
ALP: alkaline phosphatase
ALT: alanine aminotransferase
Akt: protein kinase B
ARP: actin related protein
ASA: acetyl salicylic acid
ASC-1: asc type amino acid transporter 1
AST: aspartate aminotransferase
B16BL6: mouse melanoma cell line
Bcl-2: B-cell lymphoma-2
BME: basement membrane extract
BMMs: bone marrow-derived macrophages
CAT: Catalase
CCl4: Carbon tetrachloride
CCI: chronic constriction injury
CCK8: cell counting kit-8
CDC42: cell division cycle 42
CGBVS: chemical genomics-based virtual screening
CMC: carboxy methyl cellulose
COX-2: cyclooxygenase-2
CRP: C-reaction protein
CSQ: combination of SWM and quercetinD-GAL: d-galactosamine
DAI: Disease activity index
DOI: (±)-2.5-dimethoxy-4-iodoamphetamine
DPPH: 1,1-Diphenyl-2-picrylhydrazyl radical 2, 2-Diphenyl-1-(2,4,6-trinitrophenyl) hydrazyl
DMSO: dimethylsulfoxide
DNA: deoxyriboNucleic acid
DSS: dextran sulfate sodium
ECG: electrocardiogram
ED50: 50% efficient dose
ELISA: enzyme linked immunosorbent assay
ERK: extracellular regulated protein kinase
ESR: erythrocyte sedimentation rate
FRAT1: frequently rearranged in advanced T-cell lymphomas 1
GAP: good agricultural practice
Gent: gentiopicroside
GLUT-4: glucosetransporter 4
GOT: glutamic oxaloacetic transaminase
GPx: glutathione peroxidase
GPT: glutamate pyruvate transaminase
GRD: glutathione reductase
GSH: glutathione
GSK: glycogen synthase kinase
GST: glutathione S-transferase
HCC: hepatocellular carcinoma cell
H&E: hematoxylin-eosin staining
HepG2: human hepatocellular carcinoma cells
HO-1: heme oxygenase 1
HPLC: high performance liquid chromatography
HSPC: hydrogenated soy phosphatidylcholine
Huh7: human hepatocellular cancer cell line
IC50: 50% inhibiting concentration
ICR: institute of cancer research
Ig: immune globulin
IKK: inhibitor of nuclear factor kappa B kinase
IFN: interferon
IL: interleukin
iNOS: inducible nitric oxide
ISO: isoproterenol
JAK: janus kinase
KM: Kunming
LD50: 50% lethal dose
LPS: lipopolysaccharide
L-NMMA: NG-monomethyl-l-arginine
MAF: mangiferin
MAPK: mitogen-activated protein kinase
MCF-7: human breast cancer cell line
MDA: malondialdehyde
MDA-MB-231: human breast cancer cell line
MIC: minimum inhibitory concentration
MMP: matrix metalloprotease
MPO: Myeloperoxidase
MR: Metagentiana rhodantha (Franch.) T.N.Ho and S.W.Liu
MRD: Metagentiana rhodantha (Franch.) T.N.Ho and S.W.Liu decoction
MS: mass spectrometer
MTD: maximum tolerance dose
mTOR: mammalian target of rapamycin
MTT: 3-(4,5-dimethyl-2-thiazolyl)-2,5-diphenyl-2-H-tetrazolium bromide
NBT: nitroblue tetrazolium
NCE: normochromatic erythrocyte
NIK: NF-κB inducing kinase
NF-κB: nuclear factor kB
NLRP3: nucleotide binding oligomerization domain like receptor 3
NMR: nuclear magnetic resonance
NO: nitric oxide
NQO1: NAD(P)H quinone oxidoreductase 1
Nrf2: nuclear factor E2-related factor 2
NS: normal saline
PC12: rat pheochromocytoma cell line
PCE: polychromatic erythrocytes
3T3-L1: primary mouse embryo fibroblasts
PCT: procalcitonin
PCR: quantitative real time polymerase chain reaction
PEMs: peritoneal elucidated macrophages
PGE2: prostaglandin E2
PI3K: phosphatidylinositol 3-kinase
PPAR: peroxisome proliferator-activated receptor
PWT: paw withdrawal threshold
PWL: paw withdrawal latency
RANKL: receptor activator for nuclear factor-κ B ligand
Rac1: ras-related C3 botulinum toxin substrate 1
RB: red blood cell
ROS: reactive oxygen species
RT: retention time
SAH: subarachnoid hemorrhage
SAR: structure-activity relationship
SD: spargue dawrley
SFI: sciatic functional index
SGOT: serum glutamyl oxalacetate transaminase
SGPT: serum glutamyl pyruvate transaminase
SIC: soluble immune complex
SKOV3: human ovarian carcinoma cell line
SOD: superoxide dismutase
STAT3: signal transducer and activator of transcription
STZ: streptozotocin
SWM: swertiamarin
t1/2: half-life
TBARS: thiobarbituric acid reactive substances
TBil: total bilirubin
TCM: traditional Chinese medicine
TLC: thin layer chromatography
TTC: triphenyltetrazolium chloride
TNF: tumor necrosis factor
TUNEL: TdT-mediated dUTP nick-end labeling
VC: vehicle control
WBC: white blood cell
